# Supplementary material for: Characterization of Redox-Responsive LXR-Activating Nanoparticle Formulations in Primary Mouse Macrophages
Source: Molecules. 2019 Oct 18;24(20):3751. doi: 10.3390/molecules24203751 (PMC6833070; doi:10.3390/molecules24203751)
Supplement: Supplementary file 1 [file molecules-24-03751-s001.pdf]

molecules

MDPI

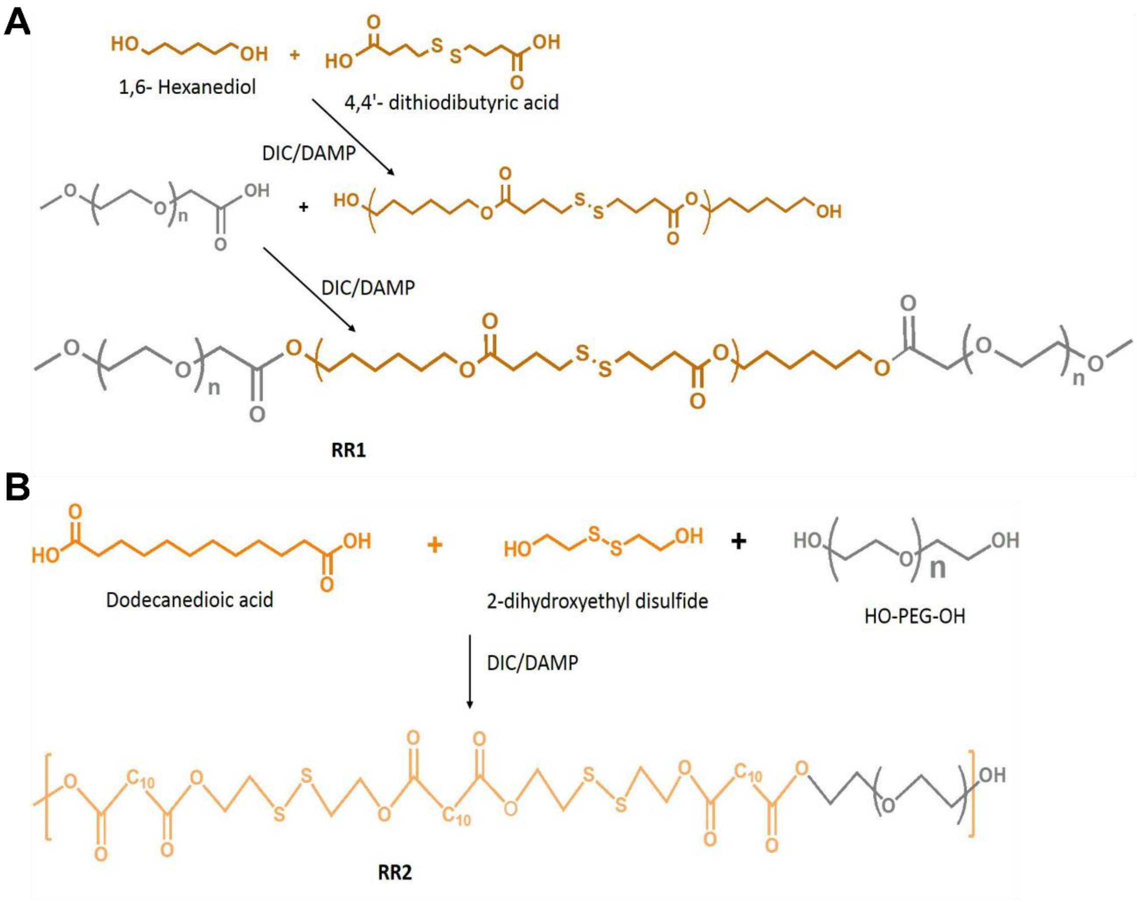

Figure S1. Schematic diagram of DB and DD chemical structures and synthesis.

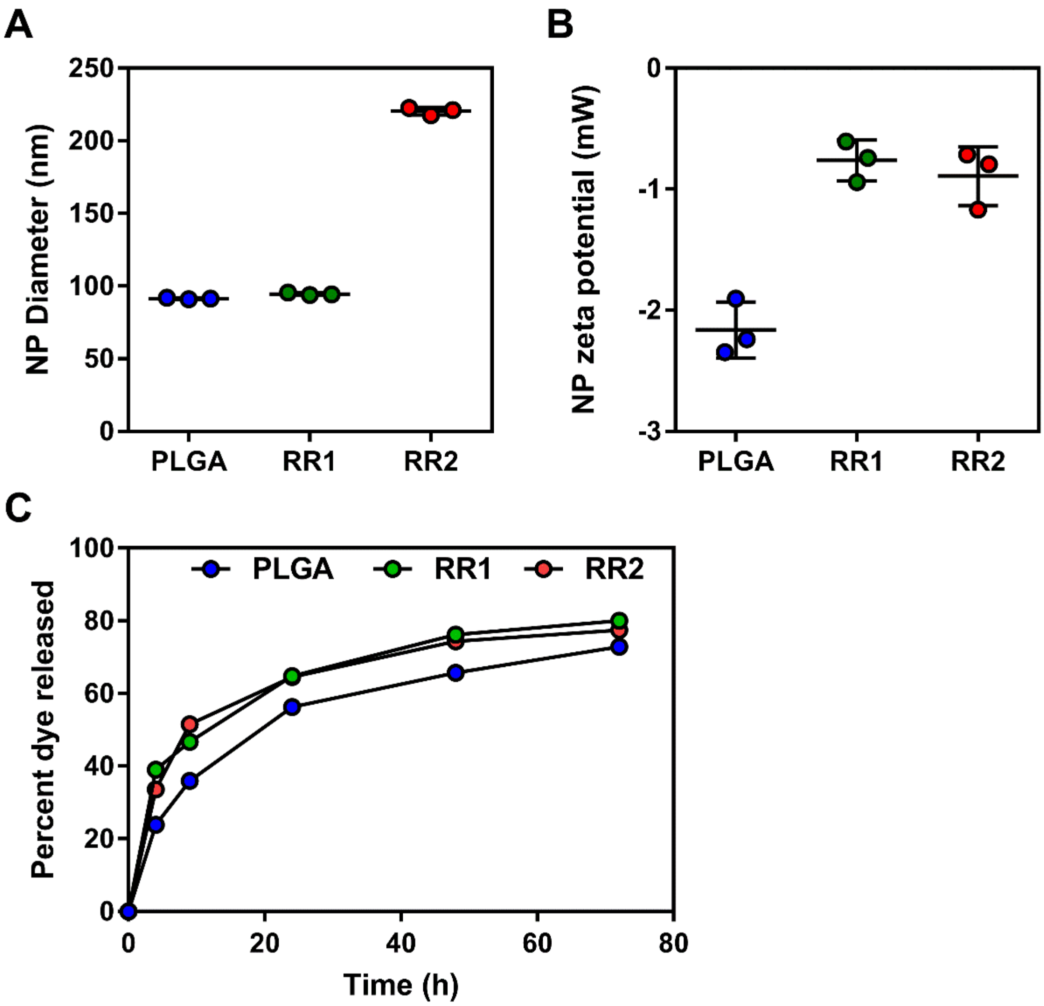

Figure S2. Empty NP characteristics. A) Particle size and B) surface charge. Each data point represents the mean of all values within each time point ± SEM (n = 3).

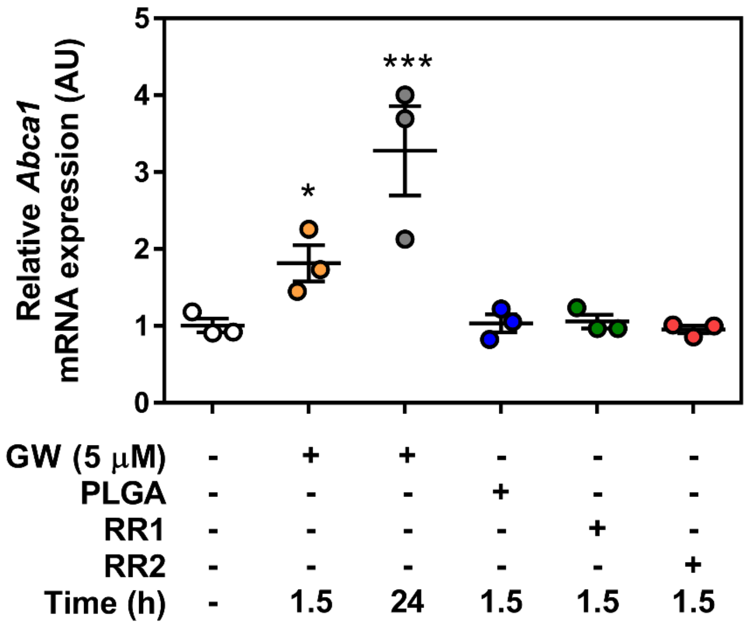

Figure S3. *Abca1* mRNA expression does not change without GW. WT BMDM were treated with empty nanoparticles or free GW drug for 1.5 h, in addition to GW (5 µg/ml) as a control for the full 24 h. *Abca1* mRNA transcript expression was measured 24 h post-empty-NP treatment by RT-qPCR. Data represent mean ± SEM where * $p < 0.05$, and ***$p < 0.001$ are differences compared with vehicle control determined by one-way ANOVA (n = 3).

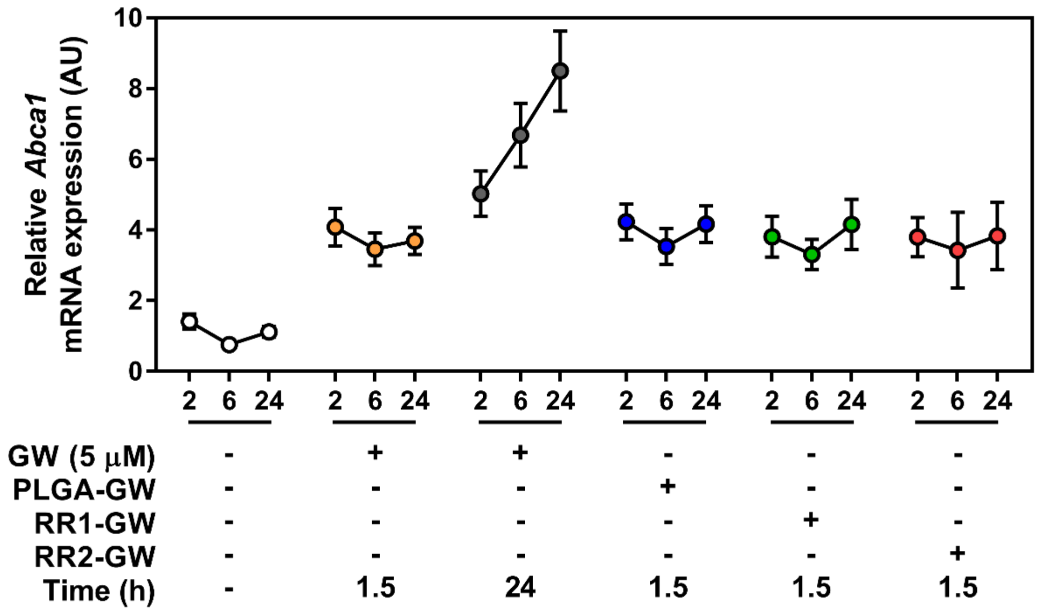

Figure S4. *Abca1* mRNA time course reveals no change in expression. Time course depicting data from Figure 4. Each data point represents the mean of all values within each time point ± SEM (n = 10-13).
